# Early Outcomes of Changes to Collection of Suicide Data in Japan

**DOI:** 10.1001/jamanetworkopen.2023.47543

**Published:** 2023-12-14

**Authors:** Nahoko Harada, Masahide Koda, Shuhei Nomura

**Affiliations:** 1 Graduate School of Interdisciplinary Science and Engineering in Health Systems, Okayama University, Okayama, Japan; 2 Co-learning Community Healthcare Re-innovation Office, Okayama University Graduate School of Medicine, Dentistry, and Pharmaceutical Sciences, Okayama, Japan; 3 Department of Health Policy and Management, School of Medicine, Keio University, Tokyo, Japan

## Abstract

This cross-sectional study examines implications of updated categorizations for suicide data collection in Japan.

## Introduction

Annually, over 700 000 suicides occur globally, with many being preventable.^1^ In Japan, the National Police Agency gathers and the Ministry of Health, Labour and Welfare publishes suicide data. In January 2022, these agencies updated their categorization, adding 23 new subcategories and modifying some existing ones.^2^ Among 7 major categories, man-woman relationships changed to interpersonal relationships, and the others (family, health, economy, work, school, and other) remained the same. Previously, up to 3 reasons were recorded for a suicide based on documents such as suicide notes. As of 2022, up to 4 reasons can be noted, including testimony of family members or others. The agencies note that comparing old and new data is not straightforward. This research examines the implications of these updates on suicide data accuracy and details.

## Methods

This cross-sectional study uses publicly available National Police Agency data.^3^ We used open data published by the Japanese government, which do not contain any personal information. The study followed the STROBE reporting guideline.

The data span from January 1, 2010, to December 31, 2022. We conducted a segmented regression analysis on the monthly suicide counts by category to gauge changes following the new data collection method starting in January 2022.^4^ Changes in the level and slope of suicide counts and 95% CIs were estimated before and after the change in data collection methodology. Adjustments were made for seasonal variations. Data were analyzed using R, version 4.3.1 (R Foundation for Statistical Computing). A 2-sided *P* < .05 was considered significant.

## Results

From January 2010 to December 2022, there were 302 439 suicide deaths with known reasons (54.7% males; 45.3% females) and 78 747 suicide deaths with unknown reasons (60.2% males; 39.8% females). There was an increase of 839 suicides with identified reasons after the data collection method change (95% CI, 639-1039). This trend was consistent across all 7 major categories. Conversely, there was a decrease of 167 suicides with unidentified reasons (95% CI, 110-225). The Figure shows the changes in trend, and the Table shows a comprehensive breakdown of the data.

**Figure.**
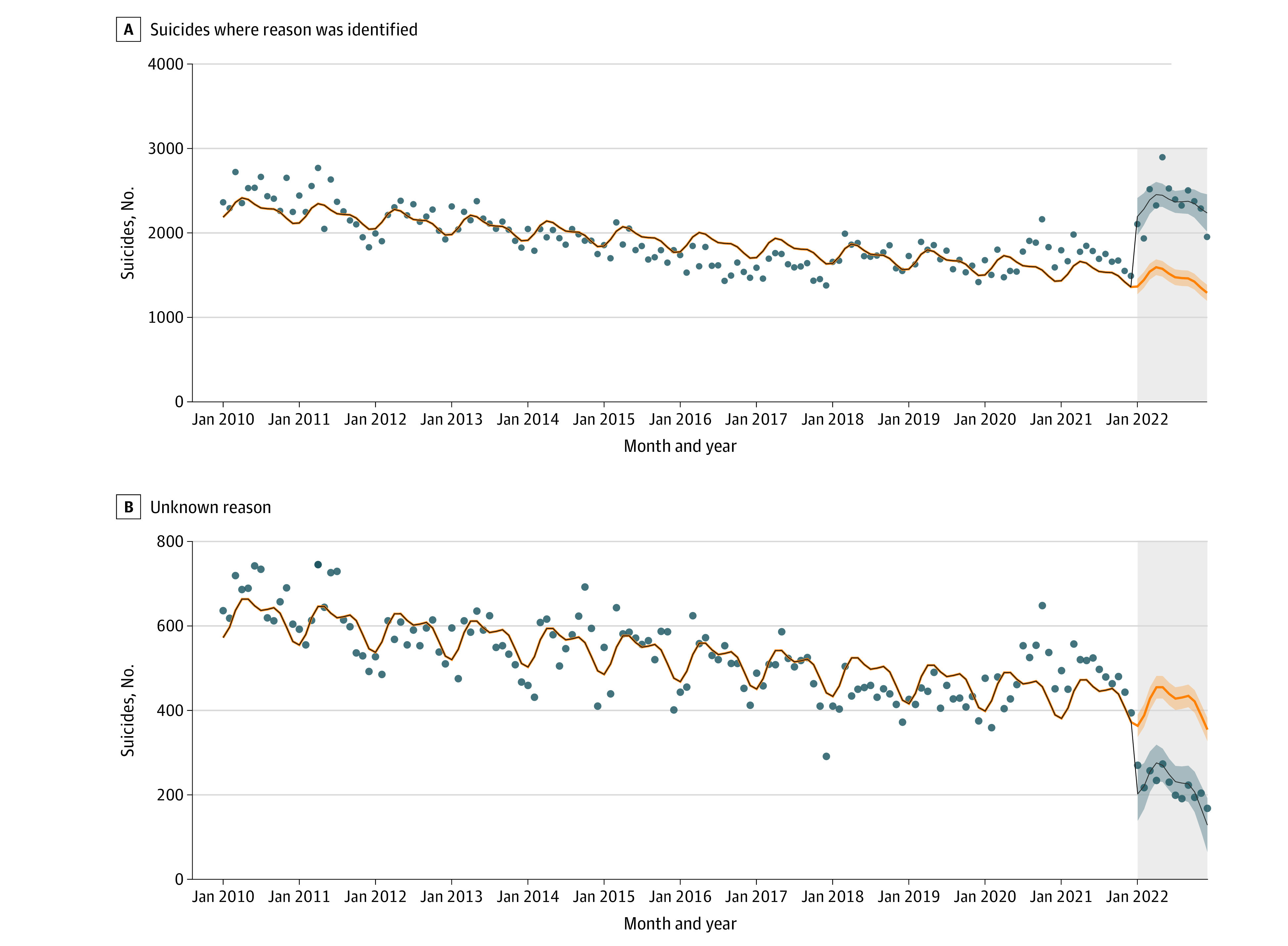
Trends in Monthly Counts of Suicides With Identified Reasons or Unknown Reasons From January 2010 to January 2022 The observational study period was from January 1, 2020, to December 31, 2021, with counterfactual associated factors determined for this period. Data in the shaded rectangle were generated starting in January 2022 using the new data collection method. The black lines represent the actual observed counts, with the shaded area depicting the 95% CI for observed data. The orange lines and their corresponding shaded areas represent the counterfactual estimates.

**Table.** Change in Levels and Slopes of Reasons for Suicide Using Linear Regression Models

Group	β (95% CI)	*P* value
Reason-identified suicides		
Level change	839 (639 to 1039)	<.001
Slope change	11 (−23 to 44)	.53
Preslope	−5.7 (−6.5 to −4.9)	<.001
Postslope	5.0 (−28.6 to 38.5)	.77
Reason		
Family		
Level change	164 (130 to 197)	<.001
Slope change	0.92 (−4.71 to 6.55)	.75
Preslope	−0.67 (−0.80 to −0.54)	<.001
Postslope	0.25 (−5.37 to 5.88)	>.93
Health		
Level change	308 (215 to 400)	<.001
Slope change	6.1 (−9.5 to 21.7)	.44
Preslope	−2.7 (−3.1 to −2.3)	<.001
Postslope	3.4 (−12.2 to 18.9)	.67
Economy		
Level change	161 (102 to 220)	<.001
Slope change	6.7 (−3.1 to 16.5)	.18
Preslope	−1.6 (−1.9 to −1.4)	<.001
Postslope	5.1 (−4.8 to 14.9)	.31
Work		
Level change	108 (83 to 132)	<.001
Slope change	−1.6 (−5.8 to 2.5)	.43
Preslope	−0.30 (−0.40 to −0.21)	<.001
Postslope	−2.0 (−6.1 to 2.2)	.35
Interpersonal relationships		
Level change	24 (12 to 36)	<.001
Slope change	−1.8 (−3.7 to 0.2)	.08
Preslope	−0.19 (−0.24 to −0.14)	<.001
Postslope	−1.9 (−3.9 to 0.0)	.06
School		
Level change	24 (17 to 31)	<.001
Slope change	−1.1 (−2.4 to 0.1)	.08
Preslope	0.0 (−0.0 to 0.0)	.80
Postslope	−1.1 (−2.4 to 0.1)	.08
Other^a^		
Level change	52 (35 to 68)	<.001
Slope change	1.5 (−1.3 to 4.3)	.29
Preslope	−0.21 (−0.28 to −0.15)	<.001
Postslope	1.3 (−1.5 to 4.1)	.36
Unknown reason		
Level change	−167 (−225 to −110)	<.001
Slope change	−5.9 (−16 to 3.8)	.23
Preslope	−1.4 (−1.7 to −1.2)	<.001
Postslope	−7.3 (−17.0 to 2.3)	.14

^a^ Other reasons include copycat suicides, survivors of crime, and criminals.

## Discussion

Our findings align with those of Inoue et al,^5^ suggesting that suicide statistics in Japan after 2021 are not straightforwardly comparable with earlier data due to changes in classifications, criteria, and the addition of new subcategories. These inconsistencies might hinder quantitative assessments and the utility of historical data for long-term strategies.

However, the changes in data collection may provide an understanding of reasons for suicide. Our findings suggest a decline in suicides with unidentified reasons. Records before January 2022 suggest that suicides not fitting within the original 52 subcategories were labeled as having unknown motives. With the addition of over 20 new subcategories and changing man-woman relationships to interpersonal relationships, a better framework exists for understanding deaths by suicide. Considering that individuals identifying as lesbian, gay, or bisexual may be twice as likely as those identifying as cisgender or heterosexual to have experienced suicidal ideations and 6 times more likely to have attempted suicide,^6^ the revised categorization may enhance reporting accuracy, which in turn could lead to tailored, data-driven suicide prevention strategies.

Another change is the inclusion of possible reasons sourced from family testimonies. While the exact motivations for suicides remain elusive, this methodological shift acknowledges the multifarious nature of suicide determinants and emphasizes the importance of formulating countermeasures grounded in a nuanced understanding. However, this new approach’s tangible benefits and practicality remain to be seen in the short term. Rigorous studies that compare data before vs after the policy shift are imperative to gauge its impact.

This study, though offering valuable insight, is based on just 1 year of data after the data collection amendment and might not encompass the long-term outcomes of the changes. However, these findings may lead to more in-depth longitudinal research on understanding suicide.

## Notes

Supplement.
Data Sharing Statement
