# Supplementary material for: Early Outcomes of Changes to Collection of Suicide Data in Japan
Source: JAMA Netw Open. 2023 Dec 14;6(12):e2347543. doi: 10.1001/jamanetworkopen.2023.47543 (PMC10722339; doi:10.1001/jamanetworkopen.2023.47543)
Supplement: Supplement. — Data Sharing Statement [file jamanetwopen-e2347543-s001.pdf]

Data Sharing Statement

Harada. Suicide Statistics in Japan. *JAMA Netw Open*. Published December 14, 2023.
doi:10.1001/jamanetworkopen.2023.47543

Data
Data available: No

Additional Information
Explanation for why data not available: Data available from Ministry of Health, Labour and Welfare. Suicide statistics: basic data on suicide in the community [Japanese]. Ministry of Health, Labour and Welfare. Accessed August 15, 2023.
https://www.mhlw.go.jp/stf/seisakunitsuite/bunya/0000140901.html
